# Treating benign ureteroenteric strictures: 27-year experience comparing endourological techniques with open surgical approach

**DOI:** 10.1007/s00345-018-2475-4

**Published:** 2018-09-19

**Authors:** M. J. van Son, M. T. W. T. Lock, M. Peters, E. E. Fransen van de Putte, R. P. Meijer

**Affiliations:** 1 Department of Radiotherapy, University Medical Center Utrecht, Heidelberglaan 100, 3584 CX Utrecht, The Netherlands; 2 Department of Urology, University Medical Center Utrecht, Utrecht, The Netherlands; 3 Department of Urology, The Netherlands Cancer Institute Antoni van Leeuwenhoek Hospital, Amsterdam, The Netherlands

**Keywords:** Urinary diversion, Anastomosis, Stenosis, Re-implantation, Endoscopy

## Abstract

**Purpose:**

To compare open surgical anastomotic revision with endourological techniques for the treatment of ureteroenteric strictures in patients with urinary diversions.

**Methods:**

All records of patients treated for ureteroenteric strictures in our clinic between 1989 and 2016 were retrospectively reviewed. In 76 patients, 161 completed procedures were analyzed: 26 open revisions vs. 135 endourological treatments, including balloon dilation, Wallstent and/or laser vaporization.

**Results:**

Median follow-up was 34 months. At 60 months, patency rates were 69% (95% CI 52–92%) after open vs. 27% (95% CI 19–39%) after endo-treatment (*p* = 0.003); median patency duration was 15.5 vs. 5 months, respectively (*p* = 0.014). Eventually, 15% of patients required open surgery after primary endo-treatment and 21% received endoscopic re-treatment after primary open surgery. Cox regression analysis revealed no confounding factors among the risk factors added to the model. Complication rates were higher after open surgery (27% Clavien 2, 12% Clavien 3–4 vs. 5% Clavien 1–2, 3% Clavien 3, *p* = 0.528). Median postoperative hospital stay was 14 days (open) vs. 2 days (endo), *p* < 0.001. Mean estimated glomerular filtration rate improved with + 17 (open) vs. + 8.1 (endo), *p* = 0.024. Renal function was compromised in 8% of patients in the open surgery group vs. 6% in the endo-treatment group.

**Conclusions:**

In these patients, in terms of patency and patency duration, open surgery was superior to endourology. Nevertheless, endourological treatments offer a safe and less-invasive alternative to delay or avoid open surgery, especially in patients who are unfit for open surgery.

## Introduction

For the construction of a urinary diversion, either a continent pouch (e.g., Indiana pouch), an orthotopic neobladder or an incontinent urostomy (e.g., ileal conduit or colon conduit) can be considered [1]. This complex reconstructive surgery is associated with significant perioperative morbidity, with reported acute complication rates of 52–78% [1–4]. Among potential long-term complications, benign obstructive ureteroenteric strictures (UES) form an important risk factor for renal function deterioration [5–7]. Although symptoms can include severe flank pain and recurrent urinary tract infections, UES can also be asymptomatic [6, 8]. Strictures consist of fibrotic tissue and are typically formed at the anastomosis between ureter and bowel segment [5, 6]. This is mostly considered to be the result of ischemia, caused by compromised vascularization during mobilization of the ureters in reconstructive surgery [5, 9, 10]. With a reported prevalence of 1.4–15% [6–9, 11–13], UES usually occur within the first 12 months after construction of the urinary diversion, although in some cases strictures may form many years postoperatively [6, 8, 12, 13]. Open surgical revision of the anastomosis has considerable risks because it is often impeded by intra-abdominal adhesions [9, 12]. Therefore, endourological techniques have been developed to potentially avoid the need for open surgery. In the available comparative literature, reported patency rates for endourology vs. open surgery are 8–29% vs. 49–95%, respectively [8, 9, 12, 13]. In our tertiary academic clinic, both endourological techniques and open surgical revision have been used to treat patients with UES. Presented here is our 27-year institutional experience with, to our knowledge, the largest cohort of UES patients analyzed until now.

## Methods

Data were retrospectively collected by reviewing the records of all patients treated for benign UES at the University Medical Center Utrecht (UMCU) between April 1989 and February 2016. The institutional Medical Research Ethics Committee provided a waiver declaring that for this type of study, formal consent was not required.

For verification of a suspected stricture after cystectomy and urinary diversion, diagnostic imaging studies included ultrasound, renal scintigraphy, retrograde loopography, antegrade pyelography and/or CT scan, depending on urologist preferences. To rule out the possibility of tumor recurrence, urine cytology was evaluated. Choice of treatment approach was mainly based on the physical condition of the patient: open surgery was preferred if the patient was fit enough. Inoperable patients were often referred to our clinic for endourologic treatment because of our extensive experience with these techniques. Endo-procedures included placement of a chronic Wallstent, laser vaporization, and balloon dilation. Excluded from the present study were: placement of a nephrostomy catheter as final or temporary treatment, temporary ureteral stent placement, and endo-procedures that were aborted due to total obstruction (10 Wallstents, 3 laser vaporizations, 2 balloon dilations).

Open surgical revision consisted of resection of the stenosed ureter and re-implantation of the patent end to the enteric segment. Follow-up imaging was performed after removal of all temporary drainage tubes such as nephrostomy tubes and catheters, with renal scintigraphy after 6 weeks, CT combined with intravenous pyelography after 12 weeks, and yearly check-ups thereafter, with renal scintigraphy alternated with CT-intravenous pyelography. Symptoms like fever or flank pain were considered indications for interim radiographic imaging. Follow-up was defined as the time between treatment and the last visit to the urology department.

Data were retrospectively analyzed for patient characteristics, patency rates and perioperative outcomes. Physical status was scored according to the American Society of Anesthesiologists (ASA) Physical Status Classification System [14]. Complications within 30 days after treatment were scored by the Clavien–Dindo Classification of Surgical Complications [15].

Primary outcomes were the patency rate after treatment and the duration of patency. Patency was defined as no radiographic sign of obstruction of the upper urinary tract, absence of infection or flank pain, and no need for nephrostomy tube placement during follow-up. Secondary outcomes were perioperative outcomes such as postoperative hospital stay, complication rate, and effect on renal function.

### Statistical analysis

Between-group differences of continuous variables were tested using the independent samples *t* test for continuous normally distributed variables, and the Mann–Whitney U test for non-normally distributed variables. Categorical data differences were tested with Pearson’s Chi-square test or Fisher’s exact test. All tests were two-sided and statistical significance was considered at *p* < 0.05.

Kaplan–Meier analysis was performed and the difference between techniques was tested with the log-rank test. For the multivariable Cox proportional hazards regression analysis, missing data for the determinants and outcome were imputed 20 times. Missing data were considered missing at random. The model was built using treatment type and adding potential confounders one by one to determine the influence of each determinant separately and in total. No categorization of continuous variables was applied to limit information loss. Baseline statistical analyses were performed using SPSS statistics version 23.0 (IBM Corp., Armonk, NY, USA). Multiple imputation (mice package [16]) and the modeling process (rms package [17]) were performed using the R language environment (version 3.2.4) [18].

## Results

Overall, 78 patients were diagnosed with UES. Two patients were excluded from the study because they opted for no treatment of their UES due to multiple sclerosis and high age, respectively. In the patient with multiple sclerosis, the kidney on the stenosed side became afunctional; the elderly patient had a renal function of 22% on the stenosed side but died of bladder cancer recurrence with metastases to the liver 18 months after diagnosis of the UES. Table 1 summarizes the baseline characteristics of 76 patients with one or two affected renal units (RU) (35 left-sided, 29 right-sided, 12 bilateral). Most patients (59%) had undergone cystectomy and urinary diversion elsewhere before they were referred to the UMCU for treatment of UES. In this study group, all patients had a refluxing end-to-side ureteroenteric anastomosis (Nesbit), with the exception of one patient who had a Wallace-I anastomosis.

**Table 1:** Baseline characteristics of the study population

	No. of patients (%)
Overall	76/88 renal units
Sex	
Male	48 (63)
Female	28 (37)
Mean age at urinary diversion in years	56 (SD 15.6)
Mean BMI at urinary diversion (range)	26 (19.4–33.8)
Reasons for urinary diversion	
Cancer	49 (65)
Severe incontinence	10 (13)
Radiation cystitis	7 (9)
Neurogenic bladder disorder	4 (5)
Bladder exstrophy	3 (4)
Interstitial cystitis	1 (1)
Unknown	2 (3)
Diversion type	
Ileal conduit	62 (82)
Indiana pouch	9 (12)
Orthotopic neobladder	4 (5)
Colon conduit	1 (1)
Stricture side per patient	
Left	35 (46)
Right	29 (38)
Bilateral	12 (16)
Stricture presentation per UES	
Asymptomatic	44 (27)
Flank pain and/or infection	61 (38)
Unknown	56 (35)
Median interval from cystectomy to diagnosis of UES in months (range)	31 (0.5–573)

*BMI* body mass index, *UES* ureteroenteric stricture

A total of 135 endourologic and 26 open surgical procedures were analyzed with a median follow-up of 34 months (range 0–319). Table 2 presents patient and stricture characteristics at the time of treatment. Overall, 88 left-sided and 73 right-sided strictures were treated. More than half of all analyzed procedures were primary procedures (53%), with a range of 1–9 procedures performed per patient. Primary endo-procedures in 72 RU were followed by one or more endoscopic re-treatments in 36%, and 15% eventually underwent open surgery. Of 14 RU primarily treated with open re-implantation, 21% required endoscopic re-treatment.

**Table 2:** Characteristics of the study population at start of treatment

	Total procedures *n* = 161 (%)	Endo *n* = 135 (%)	Open *n* = 26 (%)	*p* value
Mean age at treatment: years (range)	63 (28–84)	63.2	60.1	0.326
Mean BMI at treatment	25.9	25.4	27.4	0.099
ASA score at treatment				
I–II	66 (41)	53 (39)	13 (50)	0.279
III–IV	94 (58)	81 (60)	13 (50)	
V–VI	0 (0)	0 (0)	0 (0)	
Unknown	1 (1)	1 (1)	0 (0)	
Stricture side				
Left	88 (55)	73 (54)	15 (58)	0.734
Right	73 (45)	62 (46)	11 (42)	
Stricture length				
< 1 cm	5 (3)	5 (3)	0 (0)	0.999
> 1 cm	33 (21)	28 (21)	5 (19)	
Unknown	123 (76)	102 (76)	21 (81)	
History of pelvic radiotherapy	42 (26)	36 (27)	6 (23)	0.703
History of chemotherapy^a^	15 (9)	13 (10)	2 (8)	0.999
Primary procedure				
Yes	86 (53)	72 (53)	14 (54)	0.962
No	75 (47)	63 (47)	12 (46)	
Mean renal function of affected renal unit on renogram	53.1%	53.9%	49.8%	0.557
Mean interval from diagnosis to treatment, in months	3.1	2.8	4.6	0.142

*BMI* body mass index, *ASA* American Society of Anesthesiologists

^a^Neoadjuvant or adjuvant to cystectomy

**Fig. 1:**
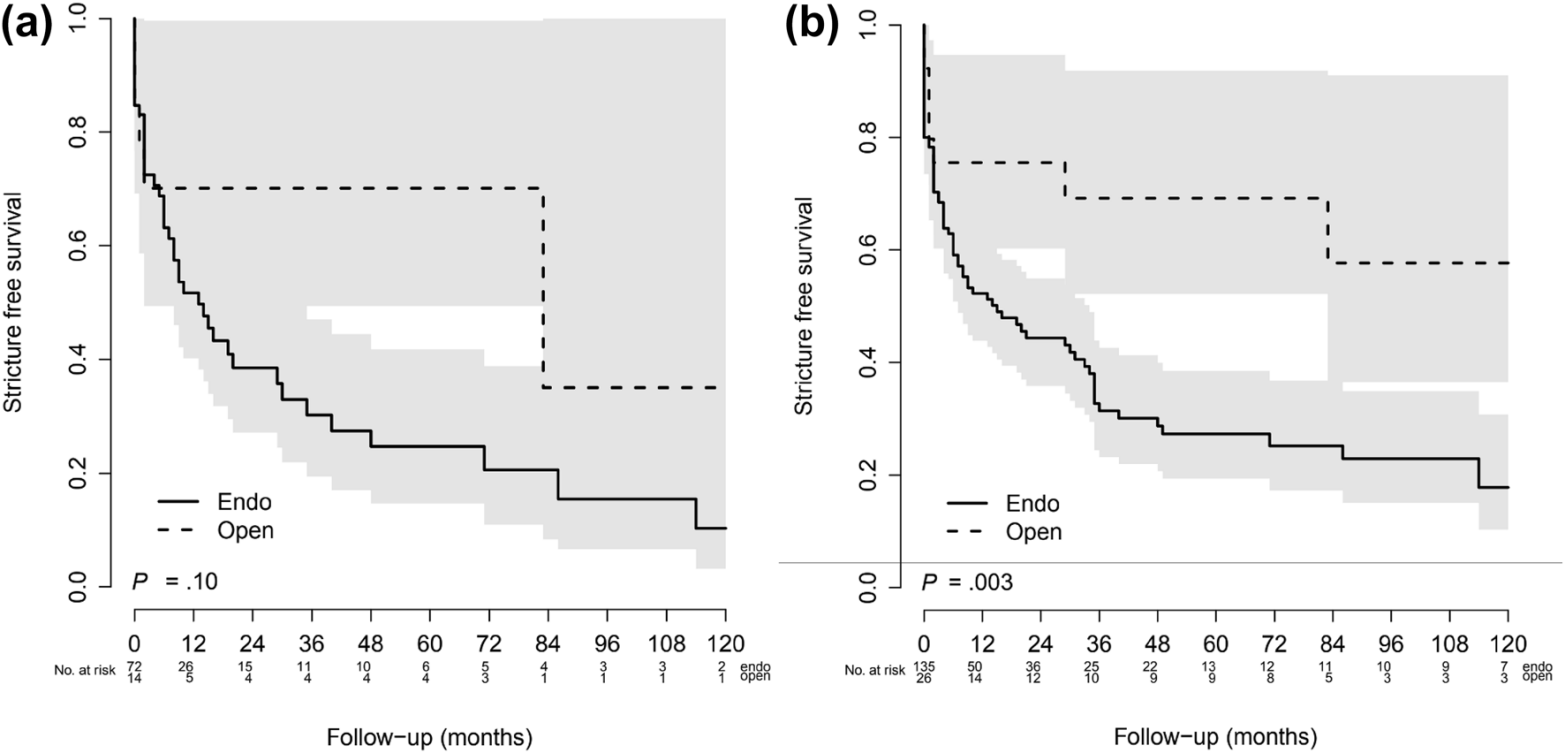
**a** Kaplan–Meier analysis for stricture-free survival after primary procedures. **b** Kaplan–Meier analysis for stricture-free survival after all procedures

Treatment results are summarized in Table 3. The absolute patency rate of 135 endo-procedures was 38.8% (42.2% for 83 Wallstents, 26.7% for 30 laser vaporizations, and 36.4% for 22 balloon dilatations) vs. 69.2% for 26 open surgical procedures (*p* value 0.003). When analyzing primary procedures only, patency rates were 38.9% after 72 endo-procedures vs. 64.3% after open surgery in 14 RU (*p* value 0.079). In the Kaplan–Meier analysis, at 60 months the patency rate after endo-procedures was 27% (95% CI 19–39%) vs. 69% (95% CI 52–92%) after open surgery. For primary procedures, at 60 months the patency rates were 25% after endo-procedures (95% CI 15–42%) vs. 70% after open surgery (95% CI 49–100%). The log-rank test revealed a significant difference when analyzing all procedures (*p* = 0.003), but not for primary procedures alone (*p* = 0.10). Kaplan–Meier curves are presented in Fig. 1a, b. Median patency duration was significantly shorter after endourological treatments compared with open surgery (5 vs. 15.5 months, *p* = 0.014).

**Table 3:** Treatment results of endourological vs. open surgical approach

	Endo (*n* = 135)	Open (*n* = 26)	*p* value
Absolute patency rate	37.8%	69.2%	0.003
Wallstent (*n* = 83)	42.2%		
Laser (*n* = 30)	26.7%		
Balloon (*n* = 22)	36.4%		
Absolute patency rate primary procedures	38.9%	64.3%	0.079
Median patency duration in months (range)	5 (1 days-256 months)	15.5 (10 days-242 months)	0.014
Number of Clavien–Dindo complications ≤ 30 days			
1	1 (1%)	0	
2	5 (4%)	7 (27%)	
3a	2 (1.5%)	0	
3b	2 (1.5%)	2 (8%)	0.528
4	0	1 (4%)	
5	0	0	
Median hospital stay in days (range)	2 (0–10)	14 (3–40)	< 0.001
Mean eGFR^a^ improvement (range)	+ 8.1 (− 3.9 + 74.4)	+17.0 (− 8.0 + 36.0)	0.024

*eGFR* estimated glomerular filtration rate

^a^Calculated with MDRD formula in mL/min/1.73 m^2^

In the endo-group, six (5%) Clavien 1–2 complications occurred, including postoperative fever requiring antibiotics, venous bleeding requiring packed cells after laser treatment and an arterial bleeding after laser treatment that was treated conservatively. Two (1.5%) Clavien 3a complications occurred, requiring intravenous antibiotics and nephrostomy catheter placement. Furthermore, two (1.5%) Clavien 3b complications occurred. A Wallstent was placed in the right ureter of a patient with an unknown Wallace-I anastomosis, resulting in obstruction of the left ureter which was treated with open re-implantation on both sides. In the other patient, laser treatment inside an obstructed Wallstent resulted in perforation of the ureter, requiring re-implantation during laparotomy. In the open group, there were seven (27%) Clavien 2 complications, including postoperative fever treated with antibiotics and wound dehiscence that was treated conservatively. Two (8%) Clavien 3b complications occurred. In one patient, acute signs of urinary leakage 7 days postoperatively resulted in re-laparotomy and reconstruction of the urinary diversion. In the other patient, the atherosclerotic arterial wall of the right common iliac artery was lacerated, requiring a femoro-femoral crossover bypass performed by a vascular surgeon. One (4%) Clavien 4 complication occurred in the open group, when a patient became septic after developing an enterocutaneous fistula and was admitted to the intensive care unit for 3 days. The enterocutaneous fistula was subsequently treated conservatively. Proportionally, there were more complications in the open surgery group (*p* = 0.528), and the complications were more severe.

Median postoperative hospital stay was significantly longer after open surgery (2 days after endo vs. 14 days after open procedures, *p* < 0.001). Mean improvement of the estimated glomerular filtration rate (eFGR) was higher after open surgery than after endo-procedures (+ 17.0 vs. + 8.1, *p* = 0.024). Renal function deterioration after treatment was seen after eight procedures (6%) in the endo-treatment group (range − 3.9 to − 0.9) and after two procedures (8%) in the open surgery group (− 8.0 and − 4.6).

Within the multivariable Cox proportional hazards regression analysis (Table 4), the chance of stricture recurrence was lower after open surgery than after endourologic treatments (HR 0.36, 95% CI 0.17–0.74, *p* = 0.006). After adjustment for risk factors (age, ASA score, BMI, history of chemotherapy/pelvic radiotherapy, renal function, stricture side and number of previous procedures), the difference in stricture recurrence risk remained significant, with minimal to no changes to the HR (HR between 0.34 and 0.36, *p* value between 0.006 and 0.009).

**Table 4:** Multivariable Cox proportional hazards regression analysis

	HR (95% CI)	*p* value
Treatment type	0.36 (0.17–0.74)	0.006
+ Age	0.36 (0.17–0.75)	0.006
+ ASA score	0.36 (0.17–0.75)	0.006
+ Body mass index	0.34 (0.16–0.74)	0.006
+ History of chemotherapy	0.35 (0.16–0.75)	0.007
+ History of pelvic radiotherapy	0.34 (0.16–0.73)	0.006
+ Kidney function	0.35 (0.16–0.76)	0.008
+ Stricture side	0.35 (0.16–0.77)	0.009
+ Primary procedure	0.35 (0.16–0.77)	0.009

*HR* hazard ratio, *95% CI* 95% confidence interval, *ASA* American Society of Anesthesiologists score

## Discussion

UES form a therapeutic challenge due to frequent recurrences and the potential risk of causing progressive loss of renal function and eventually end-stage renal disease due to obstructive nephropathy. While classic open re-implantation is related to higher complication rates and morbidity, minimally invasive endo-treatments often have poorer patency rates. In the present study, the risk of stricture recurrence was lower after open surgery, even after adjustment for patient and stricture characteristics. However, more complications occurred after open surgery and these complications were more severe. Postoperative hospital stay was significantly longer after open surgery. Both the endourologic and surgical procedures mostly improved renal function, with low rates of renal function deterioration (8% and 6%, respectively).

The available comparative literature mainly consists of small retrospective cohort studies, with heterogeneous patient and stricture characteristics [6, 8, 9, 12, 13, 19]. In these studies, different types of endo-procedures were compared with open re-implantation with absolute patency rates as their main outcome. In most studies, differences in the results between primary and secondary treatments were accounted for with reported patency rates for primary procedures of 8–29% after endo-procedures vs. 49–95% after open surgery, with a median follow-up of 21–47 months [8, 9, 12, 13]. In their analyses, Laven et al. and Tal et al. did not separate primary and secondary treatments and reported patency rates of 45–50% after endo-procedures vs. 80–93% after open surgery, at a median follow-up of 35–62.5 months [6, 19].

Patency rates vary widely between different endo-treatments. For example, DiMarco et al. reported very low patency rates of 15%, 15% and 5% at 1, 2 and 3 years, respectively, after balloon dilation (*p* = 0.0001 vs. open) [13], whereas Campschroer et al. reported that Wallstents yielded a primary patency rate of 41.1% at a mean follow-up of 37.7 months [20].

In studies focusing on renal function, open surgery does not seem superior to less-invasive or observational strategies. Helfand et al. compared long-term renal function between re-implantation and non-operative UES management, including ureteral stent or nephrostomy tube; no significant differences were found in ΔeGFR (– 25.0 vs. – 18.9 ml/min/1.73 m^2^, *p* = 0.66) or rates of renal function loss (34.6% vs. 39.5%, *p* = 0.68) [7]. Rivera et al. analyzed chronic kidney disease**-**free survival, comparing active intervention (open or endourologic treatment) with observation alone; no significant differences were found in 5-year chronic kidney disease-free survival (observation 50% vs. active treatment 33% *p* = 0.40) [21].

This comparative study has several limitations. Firstly, missing data were inevitable due to the retrospective nature of the study and the collection of data back to the year 1989. Missing data (such as ASA scores) were supplemented retrospectively based on the classification system, although misclassification might have caused information bias. Multiple imputation in the Cox model helped to reduce bias due to missing data. Within our study group, there was a propensity for right-sided strictures (45%) whereas, traditionally, UES occur more commonly on the left. Almost half of these patients (48%) were referred to us, making it difficult to explain this discrepancy. Although stricture length has been described as a risk factor [8, 13], this was not added to our model as a potential confounder because 77% of the data were missing. Also, due to a geographical incentive to return to their referring physician, some patients were lost to follow-up causing a large variability in length of follow-up. For 17.4% of all procedures, the length of follow-up was ≤ 3 months; this should be taken into account when considering the data on patency duration, which was measured until the last date of follow-up. The lack of a standardized management algorithm led to strong heterogeneity of the patient group. Over the 27 years, only 26 RU were treated with an open surgical procedure (of which 14 primarily), thereby limiting the comparative power. Confounding by indication clearly plays a role in this retrospective analysis. Differences between endourologic procedures were not fully accounted for, since the analyses mainly focused on the difference between open surgery and endourological treatments as a whole. However, chronic Wallstents yielded a higher individual success rate (42.2%). Furthermore, the retrospective analysis of renal function was obscured because measurement was not performed in a standardized manner. Therefore, the posttreatment intervals to measurement of the eGFR differed between patients and procedures.

Strengths of this study lie in the analysis of a large number of patients and procedures, facilitating a clear comparison between treatment options. Moreover, analyses were performed with an overall long period of follow-up (median 34 months, range 0–319 months). The view on treatment of UES was broadened by not only analyzing patency and patency duration, but also the complication rate, postoperative hospital stay, and effect on renal function.

Patients presenting with UES should be thoroughly counseled for their treatment options. In our opinion, there are no insurmountable disadvantages to endourological treatments, apart from the higher chance of stricture recurrence. The potential of delaying or even avoiding the need for surgical re-implantation is of great benefit, especially for patients with poor physical condition who are unfit for open surgery.

## Conclusions

In conclusion, our 27 years’ experience in treating patients with UES shows that open surgical re-implantation of the ureter results in higher patency rates with longer patency duration compared with endourological treatments. However, open surgery has higher (and more severe) complication rates and postoperative hospital stay is significantly longer. Renal function is rarely compromised by either type of treatment. Therefore, we consider endourological treatments to be a safe alternative treatment for UES, which delays or perhaps even avoids the need for open surgery.

## Abbreviations

ASA: American Society of Anesthesiologists
BMI: Body mass index
CT: Computed tomography
eGFR: estimated Glomerular filtration rate
HR: Hazard ratio
RU: Renal units
UES: Ureteroenteric stricture
UMCU: University Medical Center Utrecht
95% CI: 95% Confidence interval
